# Holistic Person-Centered Care in Radiotherapy: Protocol for a Scoping Review

**DOI:** 10.2196/51338

**Published:** 2024-04-03

**Authors:** Fatima Bhyat, Andrew Makkink, Karien Henrico

**Affiliations:** 1 Department of Medical Imaging and Radiation Sciences University of Johannesburg Johannesburg South Africa; 2 Department of Emergency Medical Care University of Johannesburg Johannesburg South Africa

**Keywords:** cancer patient, cancer, cancer care, holistic care, person-centered care, person-centered, radiologist, radiology, radiotherapist, radiotherapy, scoping review, holistic care

## Abstract

**Background:**

Several types of health care professionals are responsible for the care of patients with cancer throughout their engagement with the health care system. One such type is the radiotherapist. The radiotherapist not only administers treatment but is also directly involved with the patient during treatment. Despite this direct contact with the patient, the narrative tends to focus more on technical tasks than the actual patient. This task-focused interaction is often due to the highly sophisticated equipment and complex radiotherapy treatment processes involved. This often results in not meeting the psychosocial needs of the patient, and patients have acknowledged noncompliance and delayed treatment as a result.

**Objective:**

The scoping review aims to explore, chart, and map the available literature on holistic person-centered care in radiotherapy and to identify and present key concepts, definitions, methodologies, knowledge gaps, and evidence related to holistic person-centered care in radiotherapy.

**Methods:**

This protocol was developed using previously described methodological frameworks for scoping studies. The review will include both peer-reviewed and gray literature regarding holistic, person-centered care in radiotherapy. A comprehensive search strategy has been developed for MEDLINE (Ovid), which will be translated into the other included databases: Scopus, CINAHL (EBSCO), MEDLINE (PubMed), Embase (Elsevier), Cochrane Library, and the Directory of Open Access Journals. Gray literature searching will include Google (Google Books and Google Scholar), ProQuest, the WorldWideScience website, the OpenGrey website, and various university dissertation and thesis repositories. The title and abstract screening, full-text review, and relevant data extraction will be performed independently by all 3 reviewers using the Covidence (Veritas Health Innovation) software, which will also be used to guide the resolution of conflicts. Sources selected will be imported into ATLAS.ti (ATLAS.ti Scientific Software Development GmbH) for analysis, which will consist of content analysis, narrative analysis, and descriptive synthesis. Results will be presented using narrative, diagrammatic, and tabular formats.

**Results:**

The review is expected to identify research gaps that will inform current and future holistic, person-centered care in radiotherapy. The review commenced in November 2023, and the formal literature search was completed by the end of February 2024. Final results are expected to be published in a peer-reviewed journal by 2025.

**Conclusions:**

The findings of this review are expected to provide a wide variety of strategies aimed at providing holistic, person-centered care in radiotherapy, as well as to identify some gaps in the literature. These findings will be used to inform future studies aimed at designing, developing, evaluating, and implementing strategies toward improved holistic, person-centered care in radiotherapy.

**International Registered Report Identifier (IRRID):**

DERR1-10.2196/51338

## Introduction

Radiotherapy, also referred to as radiation therapy, is a treatment modality that uses high-energy radiation or radioactive substances to destroy cancer cells or prevent their growth [1]. The clinical usefulness of ionizing radiation was discovered after the discovery of X-rays and has since developed into a medical specialty. Ionizing radiation damages the genetic material of cancer cells, blocking the cells’ ability to proliferate, thus destroying the cancer cells [2]. Radiotherapy has become a highly cost-effective, leading modality in cancer treatment, as it can be used as a primary form of treatment or in combination with other modalities for curative or palliative purposes within cancer treatment [3]. Radiotherapy has an approximate 40% cure rate when used as a single modality of treatment compared to only 11% achieved by chemotherapy when used as a single modality of treatment [4]. Chemoradiotherapy use has experienced substantial growth and is now established as the standard treatment for various types of tumors, such as cervical, non–small cell lung cancer, and bladder cancer, as elaborated in this discussion. It provides considerable advantages in patient survival and local disease control while not significantly increasing long-term toxicities [5]. Radiotherapy is considered a conservative form of treatment with reduced adverse effects, unlike other treatments such as surgery, which can have mutilating and other long-term adverse effects, thus maintaining a good quality of life [6].

Evidence-based practice shows that more than 50% of patients with cancer will undergo a course of radiotherapy as part of their treatment, and despite this, patients appear to lack knowledge of the importance of radiotherapy treatment and its effects [7,8]. Negative perceptions toward receiving radiotherapy treatment have been expressed, as some patients with cancer consider it to be a form of poison, while others fear the effects of radiation as they believe it would cause another cancer, thus delaying seeking care [6,9]. Despite the vast improvement in radiotherapy technology, in addition to dealing with treatment-related toxicities [10], many patients also experience emotional challenges such as fear, anger, depression, stress, and anxiety [7,11]. Almost a third of patients with cancer acknowledge not having their psychosocial needs taken care of during treatment, resulting in a decrease in compliance, delayed treatment, and a decrease in quality of life, thus negatively impacting tumor control [7,12,13]. These perceptions, therefore, present an urgent need for a person-centered care approach to enhance the experience and well-being of the patient with cancer [14]. A person-centered care approach considers the needs and preferences of patients and thus allows patients to be actively involved in any treatment and decision-making [15]. Although patients with cancer come across many health professionals along their journey, radiotherapists have a key role in the experience of the patient with cancer as they find themselves in direct contact with the patient throughout the course of their radiotherapy treatment [12,16].

Radiotherapists are experts in the technical and clinical planning and delivery of radiotherapy treatment. They are equally responsible for providing care and support, monitoring and managing treatment-related toxicities, and coordinating supportive care, which can be last up to 6 weeks [8]. This places the radiotherapist in a unique position to engage with patients concerning their diagnosis and treatment [16,17]. However, despite the radiotherapist’s direct contact with the patient, the narrative tends to focus more on technical tasks and other barriers, often at the cost of important psychosocial needs not being met [18]. Radiotherapists’ disregard for patient care has also been influenced by the stressful nature of the workplace environment and the time pressures they face [17]. Nonetheless, the consequences of providing support in the form of empathy, compassion, and reassurance must be understood by radiotherapists, as it can result in alleviating their patients’ anxiety and fear before the treatment [19]. Empathy is of particular importance in cancer delivery, as patients acknowledge having greater satisfaction and self-awareness when treated with empathy [20,21]. The length of time that a patient with cancer spends interacting with a radiotherapist has been shown to have a considerable impact on their overall experience [17]. Radiotherapists’ daily direct contact with patients with cancer, therefore, not only enables them to prioritize their unique needs and values but also allows them to be involved in their care, creating a person-centered approach to treatment.

Radiotherapists can facilitate person-centered care by ensuring patients have their undivided attention [22]. This process can be initiated by building a rapport with each patient, communicating effectively, answering their questions, discussing their preferences, listening actively, addressing their physical and emotional needs, providing them with dignity, and collaborating with other health care professionals to ensure the patient’s needs are met. Patients stated that they felt more included and positive about their radiotherapy treatment when these aspects were addressed [8,17,22]. Person-centered care is also considered to be synonymous with “wholism,” or, as some may prefer, “holism.” Holistic care considers the body, mind, soul, and spirit as interrelated and acknowledges the whole experience [23]. Holistic, person-centered care is, therefore, becoming increasingly important in cancer treatment, including radiotherapy [24]. Holistic care in radiotherapy allows for a personalized approach to patient care, which includes addressing the physical, emotional, social, and spiritual needs of the patient, empowering the patient, and promoting healing and well-being, thus improving the overall quality of life of the patient.

There is a paucity of literature related to holistic, person-centered care in radiotherapy. A search of the Open Science Framework, the Cochrane Database of Systematic Reviews, and Johanna Briggs Institute (JBI) Evidence Synthesis was conducted, and no published or in progress scoping or systematic reviews were identified relating to holistic person-centered care in radiotherapy.

The proposed scoping review will explore, map, and summarize the nature, range, and extent of published literature available that relates to holistic person-centered care in radiotherapy. Identified sources will be described in terms of population, purpose, and setting, as well as theoretical underpinnings, outcome measures, and key findings. The proposed scoping review will assist in identifying common practices, knowledge gaps in the literature, and potential areas for future research and development.

## Methods

### Protocol Design

The proposed scoping review was developed using the methodological framework for scoping reviews developed by Arksey and O’Malley [25] and incorporates the updated frameworks suggested by Levac et al [26] and Peters et al [27], as well as the updated JBI methodology for scoping reviews [28]. The “population, concept, and context” (PCC) framework will guide the review, which will follow the following stages: (1) identification of the review question or questions and (2) identification of relevant studies. This will assist with the development of specific inclusion criteria to facilitate a comprehensive search of the literature. A comprehensive search for relevant studies will then be conducted, followed by the screening of those studies. The PRISMA-ScR (Preferred Reporting Items for Systematic Reviews and Meta-Analyses extension for Scoping Reviews) will guide the reporting process [29].

### Step 1: Identification of the Review Question or Questions

The research questions that will guide this scoping review are as follows: (1) What sources are available that relate to holistic, patient-centered care in radiotherapy? (2) What types of studies have been conducted on holistic, patient-centered care in radiotherapy? (3) In what settings have these studies been conducted? (4) What health care professionals and patients have been included in the studies? (5) What has the key approach been to the studies? (6) What theories have underpinned the studies? and (7) Were there any identified measures or outcomes from the studies?

### Step 2: Identification of Relevant Studies

A comprehensive search strategy has been developed by the 3 members of the research team and an experienced librarian and information specialist. An exploratory search in MEDLINE (PubMed) and CINAHL (EBSCO) was performed to identify relevant articles on the topic (Multimedia Appendix 1). Once the scoping review commences, identified keywords in the titles and abstracts of articles related to the review question will be used to complement the search strategy, thus allowing for a more comprehensive search. The search strategy will also be adapted as required by scrutinizing the reference lists of articles that meet the inclusion criteria to find additional papers. Literature published in English and studies that can be translated will be considered. The search will commence in August 2023 and will not have a date limit to ensure that all studies that meet the inclusion criteria are considered. Once the search for each database has been completed, the results will be imported into Covidence (Veritas Health Innovation) for storage, organization, removal of duplicates, screening, and mapping of the data [30].

### Step 3: Selection of Studies for Inclusion

The preliminary inclusion criteria will be defined using the PCC format. Details for each aspect of this format are described in the following sections.

#### Population

The population for this scoping review will be radiotherapists and patients involved in radiotherapy. Given that the scoping review aims to explore holistic person-centered care in radiotherapy, there will be no limitations placed relating to cancer or radiotherapy type, duration of treatment, or final outcome, nor will there be age, geographical, or time-based limitations placed on sources.

#### Concept

This review will consider studies that focus on person-centered care within radiotherapy to identify key concepts, definitions, methodologies used, existing models of care, and knowledge gaps in the literature. Any studies that included an intervention to improve person-centered care in radiotherapy and sources of evidence that informed practice will also be included.

#### Context

The scoping review will consider literature that is relevant to the radiotherapy context. The review will include the experiences of patients receiving radiotherapy treatment and evidence on the role of radiotherapists in providing any form of care or support to patients with cancer during radiotherapy treatment. in both private and public settings.

#### Types of Sources

In view of the fact that patient-centered care has been and is still a key issue in health care, the scoping review will aim to explore and integrate qualitative, quantitative, and mixed methods research data concerning person-centered care. Literature will be sourced from databases that will include Scopus, CINAHL (EBSCO), PubMed, the Education Resources Information Centre, the Cochrane Library, Sabinet, and the Directory of Open Access Journals. Any systematic reviews that meet the inclusion criteria will also be taken into account. Secondary sources that will be used in the search include Google (Google Books and Scholar), ProQuest, the WorldWideScience website, the OpenGrey website, and various university dissertations and thesis repositories.

#### Study Selection

Studies identified in the initial database search will be collated and then transferred into Covidence, which allows for duplicates to be removed and for appropriate studies to be sorted and selected based on the inclusion criteria [30]. The first stage, which involves the screening of titles and abstracts, will be performed independently by all 3 members of the research team, who will vote in Covidence to determine primary inclusion. Any disagreements will be resolved through discussion among the team members, and in the event of an impasse, the decision will be by majority vote. The second stage of article selection will involve the downloading of identified sources from the first stage and then a screening of these articles in Covidence. Full-text sources that do not meet the inclusion criteria will be recorded, and reasons for exclusion will be reported in the scoping review. Any disagreements between the research team members at any stage of the selection process will be resolved through discussion. The PRISMA-ScR checklist and flow diagram will be used to report and present in full the results of the search and study inclusion selection in the final scoping review [28,29].

### Step 4: Charting and Extraction of the Data

The extraction tool will be developed by the research team (Multimedia Appendices 2 and 3) in accordance with the JBI methodology for scoping reviews [29] and will be piloted in Excel (Microsoft Corporation) for relevance and ease of use. With the aid of an extraction tool, data will be independently extracted from the selected studies by the 3 members of the research team. Covidence has the advantage that changes can be made to the data extraction form at any time, even while extraction is in progress [30]. The data extracted will include study-specific details regarding article title, authors, geographic area, journal or other source, year of publication, aim or purpose, study population and sample size, context, methodology, intervention, outcomes, and key findings related to the scoping review questions. Data must be compared, and a consensus must be reached before the data can be exported. After completing the literature search and collecting all related evidence regarding person-centered care in radiotherapy, the next step will be to chart the data. This step involves synthesizing and interpreting the data, which allows key issues and themes to be highlighted. Sources will be imported into ATLAS.ti (ATLAS.ti Scientific Software Development GmbH), and thematic analysis will be used to identify themes that will then be summarized and discussed in relation to the study aims and objectives [31]. The identified codes and themes will be used to map the different aspects of person-centered care in radiotherapy as well as any evidence gaps. Should any missing data be noted in the data extraction table, it will be noted as “missing.” The data will be collated from ATLAS.ti and presented in a narrative summary and table format.

### Step 5: Collation, Synthesis, and Reporting of the Results

The results will allow the researchers to better understand the delivery of holistic, person-centered care in radiotherapy. In addition, the role of the radiotherapist will be analyzed, as will what is required of them to enhance this phenomenon. The results may also raise awareness of important issues in the radiotherapy profession that should be included in the education of future radiotherapists. The results will be reported in accordance with the PRISMA-ScR reporting guidelines.

## Results

The scoping review commenced in November 2023, and the formal literature search was completed by the end of February 2024. We are currently in the process of screening articles. A preliminary search of the literature was conducted using the Scopus database in November 2023, revealing 37,345 results using the initial search terms. We intend to publish the manuscript in an appropriate, peer-reviewed, and open-access health care journal to describe our findings and conclusions to the academic audience in 2025.

## Discussion

### Overview

There are several aspects related to patient-centered care within radiotherapy where gaps exist, but these are not well described. This scoping review intends to provide an overview of the current knowledge related to patient-centered care in radiotherapy, the effects that patient-centered care in radiotherapy has on both patients and radiotherapists, and to highlight existing gaps in the literature. The use of gray literature may also supplement the search to find pertinent information that cannot be found in the databases. Finally, this strategy will provide an important lens for understanding the value of delivering holistic, person-centered care in radiotherapy. The scoping review method complies with the JBI recommendations [28] and will use the most recent reporting guidelines [29] to ensure that a comprehensive process that can be tracked is used.

The findings will assist radiotherapists and radiotherapy departments, as well as educational institutions, in identifying elements that are vital for the delivery of holistic, person-centered care in radiotherapy. The study may also determine the need for additional curriculum content or additional courses for radiotherapists to fulfill their role as holistic care providers.

### Limitations

The scoping review has some limitations. First, the search terms are variable due to the differing terminology used to describe radiotherapy and patients with cancer. Also, the research team will only consider sources available in English, meaning that sources not written in English will be missed, limiting generalizability. The quality of the studies included will not be evaluated for quality or methodological rigor; however, this is acceptable within the context of a scoping review and the aims of this review.

### Conclusion

The scoping review will assist in providing information that will be indispensable in the development of a conceptual framework to guide holistic, person-centered care in radiotherapy. The results will be disseminated at a national radiotherapy conference, including publishing a paper in a radiotherapy journal. This scoping review strategy hopes to address a gap in the literature and create awareness for radiotherapists about the importance of delivering holistic, person-centered care.

## Appendix

Multimedia Appendix 1 Search terms and results from an initial search conducted in Scopus.

Multimedia Appendix 2 JBI template source of evidence details, characteristics, and results extraction instrument.

Multimedia Appendix 3 Data extraction sheet developed by researchers for data extraction.

## Abbreviations

JBI: Johanna Briggs Institute
PCC: population, concept, and context
PRISMA-ScR: Preferred Reporting Items for Systematic reviews and Meta-Analyses extension for Scoping Reviews
