# Supplementary material for: Holistic Person-Centered Care in Radiotherapy: Protocol for a Scoping Review
Source: JMIR Res Protoc. 2024 Apr 3;13:e51338. doi: 10.2196/51338 (PMC11024745; doi:10.2196/51338)
Supplement: Multimedia Appendix 1 [file resprot_v13i1e51338_app1.docx]

**Tabe S1**. Search terms and results from an initial search conducted in Scopus.

| **PRIMARY TERM** | **STRING NUMBER** | **TERMS** | **RESULTS** |
| --- | --- | --- | --- |
| Concept 1: Radiotherapy | #R1 | radiotherapy | 496,675 |
|  | #R2 | radiation therapy | 301,853 |
|  | #R3 | radiotherapist | 1,864 |
|  | #R4 | radiation therapist | 1,654 |
|  | #R5 | cancer | 3,962,725 |
|  | #R6 | oncology | 253,967 |
|  | #R7 | oncologist | 35,460 |
|  | #R8 | therapeutic radiography | 29,400 |
|  | #R9 | radiation treatment | 379,887 |
|  | #R10 | #R1 OR #R2 OR #R3 OR #R4 OR #R5 OR #R6 OR #R7 OR #R8 #R9 | 4,153,735 |
| Concept 2:  patient-centred | #P1 | ‘person-centred’ | 15,962 |
|  | #P2 | ‘person centred’ | 25,511 |
|  | #P3 | ‘person centric’ | 1,602 |
|  | #P4 | ‘person focussed’ | 25,902 |
|  | #P5 | ‘patient-centred’ | 53,103 |
|  | #P6 | ‘patient centred’ | 83,705 |
|  | #P7 | ‘patient centric’ | 8,292 |
|  | #P8 | ‘patient focussed’ | 151,876 |
|  | #P9 | ‘relationship-centred’ | 682 |
|  | #P10 | ‘relationship centred’ | 33,852 |
|  | #P11 | ‘client centred’ | 7,512 |
|  | #P12 | holistic | 144,430 |
|  | #P13 | wholistic | 835 |
|  | #P14 | psychosocial | 192,420 |
|  | #P15 | #P1 OR #P2 OR #P3 OR #P4 OR #P5 OR #P6 OR #P7 OR #P8 OR #P9 OR #P10 OR #P11 OR #P12 OR #P13 OR #P14 | 433,174 |
| Concept 3:  care | #C1 | care | 4,574,313 |
|  | #C2 | caring | 83,549 |
|  | #C3 | treatment | 9,956,615 |
|  | #C4 | well-being | 267,128 |
|  | #C5 | therapeutic | 1,759,539 |
|  | #C6 | therapy | 5,407,278 |
|  | #C7 | support* | 6,617,069 |
|  | #C8 | healthcare | 600,092 |
|  | #C9 | health care | 2,678,706 |
|  | #C10 | #C1 OR #C2 OR #C3 OR #C4 OR #C5 R #C6 OR #C7 OR #C8 OR C9 | 21,621,180 |
| Final search string |  | #R10 AND #P15 AND #C10 | 37,345 |
