# Supplementary material for: Holistic Person-Centered Care in Radiotherapy: Protocol for a Scoping Review
Source: JMIR Res Protoc. 2024 Apr 3;13:e51338. doi: 10.2196/51338 (PMC11024745; doi:10.2196/51338)
Supplement: Multimedia Appendix 2 [file resprot_v13i1e51338_app2.docx]

**Table S1**. Johanna Briggs Institute template source of evidence details, characteristics, and results extraction instrument.

| **Scoping Review Details** |  | |
| --- | --- | --- |
| Scoping Review title: |  | |
| Review objective/s: |  | |
| Review question/s: |  | |
| **Inclusion/Exclusion Criteria** |  | |
| Population |  | |
| Concept |  | |
| Context |  | |
| Types of evidence source |  | |
| **Evidence source Details and Characteristics** | |  |
| Citation details (e.g., author/s, date, title, journal, volume, issue, pages) |  | |
| Country |  | |
| Context |  | |
| Participants (details e.g., age/sec and number) |  | |
| **Details/Results extracted from source of evidence** [in relation to the concept of the scoping review] | | |
| E.g., Quality of Life Domains assessed |  | |
| E.g., Number of items in tool |  | |
| E.g., Details of psychometric validation of tool |  | |
