# Supplementary material for: Holistic Person-Centered Care in Radiotherapy: Protocol for a Scoping Review
Source: JMIR Res Protoc. 2024 Apr 3;13:e51338. doi: 10.2196/51338 (PMC11024745; doi:10.2196/51338)
Supplement: Multimedia Appendix 3 [file resprot_v13i1e51338_app3.docx]

**Table S1**. Johanna Briggs Institute template source of evidence details, characteristics, and results extraction instrument.

| Article Title | Author(s) | Journal or Other Source | Year of Publication | Origin/Country of Origin | Aims/  Purpose | Study Population and Sample Size | Context | Methodology/  Methods | Intervention Type/Duration, Comparator, Outcome Measures | Outcomes | Key Findings that Relate to the Scoping Review Question/s |
| --- | --- | --- | --- | --- | --- | --- | --- | --- | --- | --- | --- |
|  |  |  |  |  |  |  |  |  |  |  |  |
|  |  |  |  |  |  |  |  |  |  |  |  |
